# Advances in cancer research on FUT8 molecular mechanisms and clinical applications

**DOI:** 10.1097/JS9.0000000000002669

**Published:** 2025-06-10

**Authors:** Qiancheng Ma, Yan Liu, Qing Fan, Xinqing Zhu, Deyong Yang, Ziqi Zhao

**Affiliations:** a First Affiliated Hospital of Dalian Medical University, Dalian, Liaoning, China; b Department of Surgery, Healinghands Clinic, Dalian, Liaoning, China

**Keywords:** biomarker, FUT8, immunity, molecular mechanism, tumor

## Abstract

Fucosyltransferase 8 (FUT8) catalyzes core fucosylation, a critical post-translational modification influencing cellular processes. Aberrant core fucosylation is linked to tumor progression, including proliferation, metastasis, and immune evasion. This review integrates the transcriptional and post-transcriptional regulation of FUT8, its interactions with key signaling molecules (E-cadherin, epidermal growth factor receptor, TGF-β, and β-catenin), and their impact on tumor behavior. We evaluate FUT8’s role in cancer immunity, focusing on antibody-dependent cell-mediated cytotoxicity and immune checkpoint regulation, alongside the development of FUT8 inhibitors for clinical use. Additionally, we explore FUT8 and core fucosylation products as diagnostic and prognostic biomarkers. By analyzing molecular mechanisms, immune interactions, and clinical potential, we highlight FUT8’s pivotal role in cancer biology, address its tissue-specific pro- and anti-tumorigenic roles, and propose future research to enhance cancer diagnosis, treatment, and prognosis.

## Introduction

**Figure 1.**
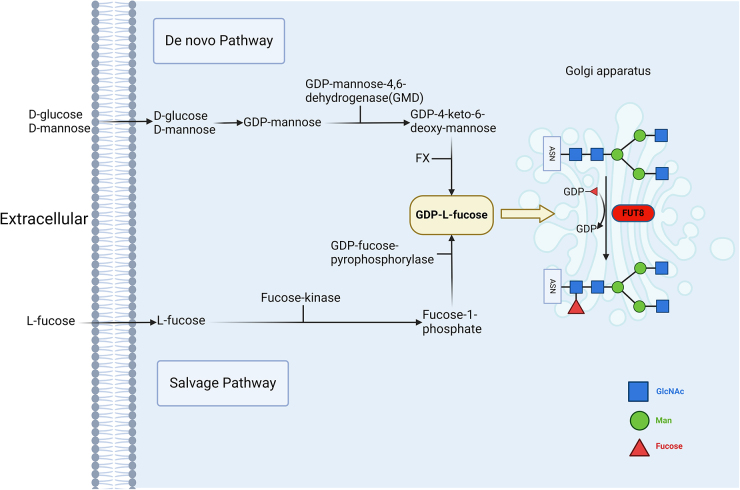
Synthesis of GDP-l-fucose and the role of FUT8 in core fucosylation. GDP-l-fucose, the donor substrate for FUT8, is synthesized via two pathways: the de novo pathway and the salvage pathway. In the de novo pathway, d-glucose or d-mannose undergoes a series of enzymatic reactions to first produce GDP-mannose, which is then converted to GDP-l-fucose by the combined actions of GDP-mannose-4,6-dehydrogenase (GMD) and GDP-keto-6-deoxymannose 3,5-epimerase,4-reductase (also known as the FX protein). In the salvage pathway, free l-fucose from extracellular sources is recycled and transformed into GDP-l-fucose through the action of fucose-kinase and GDP-fucose-pyrophosphorylase. Once GDP-l-fucose is synthesized, it is transferred by FUT8 in the Golgi apparatus to the innermost GlcNAc (*N*-acetylglucosamine) residue of an *N*-glycan, forming an α-1,6 linkage, a process known as core fucosylation. This differs from terminal fucosylation, where fucose is added to the ends of glycan chains by other fucosyltransferases. Created with BioRender.Com. FUT8, fucosyltransferase 8; FX, GDP-keto-6-deoxymannose 3,5-epimerase,4-reductase; GMD, GDP-mannose-4,6-dehydrogenase.

Glycosylation, the enzymatic addition of monosaccharides to carbohydrates, proteins, or lipids via glycosidic bonds, is among the most intricate and diverse post-translational modifications in living organisms^[1,2]^. Protein glycosylation mediates immune recognition, cell adhesion, and signal transduction and is closely associated with cancer cell proliferation, invasion, and migration^[3-9]^. *N*-Glycosylation and *O*-glycosylation are the two main forms of protein glycosylation. Fucosylation, a critical step in the formation of certain *N*-glycans, *O*-glycans, and glycolipids, involves the transfer of l-fucose from GDP-l-fucose to glycan chains by fucosyltransferases (FUTs)^[10]^. The synthesis of GDP-l-fucose proceeds via two pathways: the de novo pathway and salvage pathway^[10-12]^. In the de novo pathway, GDP-l-fucose is synthesized from GDP-mannose through the action of GDP-mannose-4,6-dehydratase (GMD) and GDP-keto-6-deoxymannose 3,5-epimerase,4-reductase (also known as FX protein), which accounts for approximately 90% of the cytosolic GDP-l-fucose production^[13-15]^. In the salvage pathway, L-fucose is converted to GDP-l-fucose via fucose-kinase and GDP-fucose pyrophosphorylase^[16,17]^ (Fig. 1). Fucosylation can be classified into core fucosylation and terminal fucosylation, depending on the position of the glycosidic bond within the *N*-glycan^[18]^. Among the 13 identified fucosyltransferases (FUT1–FUT13), 11 (FUT1–FUT11) catalyze the formation of fucosyl bonds in the Golgi apparatus^[19]^. POFUT1 (FUT12) and POFUT2 (FUT13) directly add fucose to serine/threonine residues in the endoplasmic reticulum, contributing to *O*-glycan formation^[19,20]^. Fucosyltransferase 8 (FUT8) adds l-fucose to the GlcNAc residue at the core of the *N*-glycans through an α-1,6 bond. As the sole glycosyltransferase responsible for catalyzing core fucosylation, FUT8 has garnered significant attention.

**HIGHLIGHTS**

- Comprehensive integration of FUT8 regulatory networks and its functions in key signaling pathways.
- Systematic overview of FUT8’s roles in tumor immunity and its therapeutic value.
- Discussion of FUT8’s potential as a biomarker.
- First exploration of the tissue-specific dual roles of FUT8 across different cancer types.
- Summary of unresolved mechanistic questions and future directions for clinical translation.

FUT8 is located on chromosome 14q23.3, spanning over 50 kb of the genomic region and encompassing at least 9 exons and 387,280 base pairs. The coding sequence of FUT8 consists of eight exons. The 5ʹ flanking region of exon 1 exhibits promoter activity and includes potential transcription factor (TF)-binding sites, such as bHLH, c-Myb, GATA-1, and a TATA box. The translation initiation codon is found in exon 2^[21,22]^. FUT8 comprises multiple domains, including an N-terminal cytoplasmic region, transmembrane region, stem region, coiled-coil domain, catalytic domain, and Src homology 3 (SH3) domain^[23,24]^. The coiled-coil domain contributes to FUT8 oligomerization and enzymatic activity^[25]^. As the only glycosyltransferase containing an SH3 domain, the SH3 domain of FUT8 enhances its enzymatic activity and regulates its localization^[24]^. The stem region plays a critical role in the intracellular localization of FUT8, as mutants lacking this region show significantly reduced localization in the Golgi apparatus^[26]^.

As one of the most critical modifications in *N*-glycan processing, the abundance, activity, and localization of FUT8 in the Golgi apparatus directly influence the expression of core fucosylation^[8]^. FUT8 plays an essential role in physiological processes, as Fut8 knockout mice exhibit severe growth retardation, emphysema-like changes, and perinatal lethality^[27]^. Recent studies have shown that abnormal FUT8 expression is closely associated with the progression of various cancers^[28]^. These include upregulation in hepatocellular carcinoma (HCC), clear cell renal cell carcinoma (ccRCC), melanoma, non-small cell lung cancer (NSCLC), pancreatic cancer, and colorectal cancer (CRC), and downregulation in gastric cancer, osteosarcoma (OS), and cervical squamous cell carcinoma^[29-37]^. Further research on FUT8 may provide valuable insights into cancer biology.

In this review, we provide a comprehensive and up-to-date overview of the role of FUT8 in cancer biology. Addressing several critical gaps in the current literature, we systematically summarize the transcriptional and post-transcriptional regulation of FUT8, its interactions with major oncogenic signaling pathways, and its immunological functions in modulating antibody-dependent cell-mediated cytotoxicity (ADCC) and immune checkpoint stability. We further explore the emerging therapeutic potential of FUT8 inhibitors and afucosylated antibodies, as well as the diagnostic and prognostic value of fucosylated proteins and FUT8 expression. Notably, we discuss for the first time the tissue-specific dual roles of FUT8 across different tumor types. Unresolved mechanistic questions and future directions for clinical translation are also highlighted. This review aims to provide researchers with a clear framework for understanding the multifaceted roles of FUT8 in cancer and to inspire novel studies and therapeutic strategies. Recent guidelines emphasize the importance of transparency in AI-assisted manuscript preparation^[38]^. In the present review, AI was used as a reference for translating some sentences, with all final content reviewed and confirmed by all authors.

## Regulation of FUT8 expression in cancer

Advancements in sequencing technologies have identified multiple factors regulating FUT8 at transcriptional and post-transcriptional levels, influencing its role in cancer. This section summarizes these regulatory mechanisms, categorized by transcriptional and post-transcriptional processes, and their implications for tumor biology (Table 1).

**Table 1:** Regulation factors of FUT8 expression at the transcriptional and post-transcriptional level

Type of factor	Function	Factor	Tumor	Related Mechanism	References
TFs	Initiating the transcription of the FUT8 gene.	TGIF2	Melanoma	–	^[31]^
		STAT3	BC, HCC	In BC, miR-10b binds to 3ʹ-UTR of AP-2γ gene and inhibits STAT3 phosphorylation. In HCC, HOTAIR recruits P300 and STAT3 to form a transcriptional complex	^[39-41]^
		TCF/LEF	HCC, NSCLC	In HCC, Cav-1 activates the Wnt/β-catenin pathway and promotes TCF/LEF binding to the FUT8 promoter	^[32,42]^
		p53	HCC, CRC	In HCC, acetylation-mediated activation of p53 by HDACi upregulates FUT8 transcription	^[43,44]^
miRNA	Inhibiting the translation of FUT8 mRNA.	miR-34a/miR-26a/miR-455-3p	HCC	In HCC, miR-34a, miR-26a, and miR-455-3p bind to the FUT8 3′-UTR and inhibit its expression.	^[29]^
		miR-198	CRC, NSCLC	In HCC, miR-34a, miR-26a, and miR-455-3p bind to the FUT8 3′-UTR and inhibit its expression. In NSCLC, miR-198-5p targets 3′-UTR of FUT8 mRNA	^[45,46]^
		miR-186	OSCC, LUAD	In OSCC and LUAD, miR-186 targets and inhibits the transcription of FUT8 mRNA	^[47,48]^
		miR-122-5p	CRC, iCCA	In CRC, Fn activates the TGF-β1/Smads signaling pathway by regulation of the miR-122-5p/FUT8 axis	^[49,50]^
		miRNA-1275	Cervical cancer	RACK1 inhibits miR-1275, which increases FUT8 mRNA expression	^[51]^
CircRNA	Attenuating its inhibitory effect by binding to miRNA.	cFUT8	HCC, LUAD	In HCC, cFUT8 acts as a sponge for miR-548c, disrupting the miR-548c/FUT8 regulatory axis. In LUAD, cFUT8 competitively binds YTHDF2 and miR-186-5p to stabilize FUT8 mRNA	^[52]^
					^[48]^
LncRNA	Indirect effect	LEF-AS1	CRC	LEF-AS1 recruits MLL1 to the LEF1 promoter, upregulating LEF1 transcription and consequently increasing FUT8 expression	^[34]^
		SNHG1	OSCC	In OSCC, SNHG1 binds to miR-186 and upregulates FUT8 level	^[47]^

BC, breast cancer; circRNAs, circular RNAs; CRC, colorectal cancer; Fn, Fusobacterium nucleatum; FUT8, fucosyltransferase 8; HCC, hepatocellular carcinoma; HDACi, histone deacetylase inhibitor; lncRNAs, long noncoding RNAs; LUAD, lung adenocarcinoma; miRNAs, microRNAs; NSCLC, non-small cell lung cancer; OSCC, oral squamous cell carcinoma; RACK1, receiver for Activated C Kinase 1; TCF/LEF, T-cell factor/lymphoid enhancer-binding factor; TFs, transcription factors.

### Transcriptional regulation

TFs initiate FUT8 transcription, modulating core fucosylation levels. TGIF2 positively regulates FUT8 transcription by binding to its promoter in melanoma cells^[31]^. In breast cancer (BC) cells, the TF activating protein 2γ (AP-2γ) interacts with STAT3 to reduce the production of p-STAT3, thereby inhibiting the nuclear translocation of p-STAT3 to activate FUT8 transcription^[39]^. LEF1, a gene encoding TCF/LEF proteins, facilitates the transcriptional activation of FUT8 in CRC^[34]^. p53, a critical tumor suppressor gene, is frequently lost or mutated during cancer development. Studies have shown that the p53 protein can also act as a TF to promote FUT8 expression. In HCC, wild-type p53 binds to the FUT8 promoter region to enhance transcription^[43]^. Research by Masaru Noda revealed that in CRC, FUT8 mRNA expression is significantly higher in tumors with wild-type p53 than in those with mutant p53, and that the prognostic value of FUT8 is associated with p53 status^[44]^. However, the specific prognostic relationship between FUT8 and p53 requires further exploration of the underlying molecular mechanisms.

### Post-transcriptional regulation

Noncoding RNAs (ncRNAs) are functional RNA molecules whose roles in cancer are gradually being uncovered. Current evidence has confirmed that certain microRNAs (miRNAs), circular RNAs (circRNAs), and long noncoding RNAs (lncRNAs) are closely associated with the transcription and translation of FUT8^[28]^.

miRNAs are ncRNAs approximately 20–24 nucleotides in length that inhibit mRNA translation by binding to the 3ʹ-UTR region. Several miRNAs that interact with FUT8 mRNA have been identified. Notably, miR-34a, miR-26a, miR-455-3p, and miR-548c have been found to suppress FUT8 expression in HCC^[29,52]^. In CRC, miR-198 downregulation promotes cell proliferation and metastasis^[45]^. Similarly, in NSCLC, miR-198-5p downregulation indirectly enhances cancer cell migration, invasion, and epithelial–mesenchymal transition (EMT)^[46]^. miR-186 suppresses malignancy in oral squamous cell carcinoma (OSCC) and lung adenocarcinoma (LUAD)^[47,48]^. Additionally, miR-122-5p inhibits FUT8 mRNA expression in intrahepatic cholangiocarcinoma (iCCA) and CRC, and its downregulation significantly increases proliferation and migration in both cancers^[49,50]^. Recent studies indicate that the Receiver for Activated C Kinase 1 (RACK1) promotes FUT8 mRNA expression in cervical cancer cells by inhibiting miRNA-1275^[51]^. Across these cancer types, increased malignancy is closely related to miRNA downregulation, highlighting the critical role of miRNA-mediated suppression of FUT8 mRNA in cancer progression.

CircRNAs are formed by back-splicing, which links the 5ʹ and 3ʹ ends of the linear RNA. Existing studies have revealed the molecular mechanisms of circRNA cFUT8 in certain cancers, where it acts as an “miRNA sponge” to block miRNA interference with FUT8 mRNA. Chong Li *et al* found that cFUT8 is highly expressed in HCC and binds to free miR-548c, indirectly promoting HCC proliferation, invasion, and malignancy^[52]^. Dong G *et al* demonstrated that m6A-modified cFUT8 binds to miR-186-5p and YTHDF2, preventing FUT8 mRNA degradation and thereby enhancing LUAD malignancy^[48]^.

LncRNAs typically exceed 200 nucleotides in length and are not translated into proteins. Research has increasingly demonstrated their significant roles in tumorigenesis and metastasis^[53]^. In CRC, LEF1-AS1 recruits MLL1 to the LEF1 promoter region, indirectly regulating FUT8 via enhanced LEF1 transcription^[34]^. SNHG1 upregulates FUT8 mRNA expression by binding to miR-186^[47]^. Furthermore, FUT8 generates FUT8-AS1 through reverse transcription. In OSCC, upregulated FUT8-AS1 activates the Wnt/β-catenin pathway, promoting tumor proliferation and migration^[54]^. Conversely, in melanoma, FUT8-AS1 downregulation inhibits the NRAS/MAPK signaling pathway, thereby suppressing cancer cell proliferation, migration, and invasion^[55]^. Although research on lncRNAs is relatively limited, existing studies suggest that lncRNAs indirectly regulate FUT8 transcription and translation and that FUT8-AS1 is closely related to tumor biological behavior.

## Interactions of FUT8 with cancer-related signaling pathways

**Figure 2.**
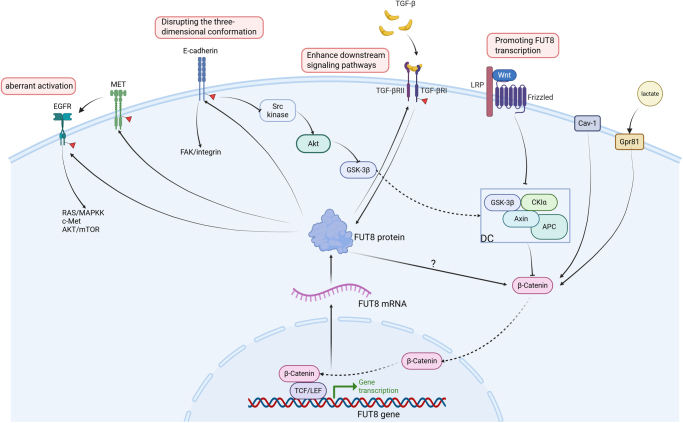
FUT8-mediated modulation of cancer-related signaling pathways. FUT8 promotes core fucosylation of E-cadherin, altering its three-dimensional conformation. This modification inhibits Src kinase activation and enhances FAK/integrin signaling, thereby modulating cancer cell behavior, while simultaneously suppressing the Src–Akt–GSK-3β pathway. Core fucosylation of EGFR results in its aberrant activation, potentially facilitating the transmission of multiple oncogenic pathways, including Ras/MAPKK, c-Met, and Akt/mTOR. Additionally, FUT8 can induce transactivation of EGFR via the HGF/MET pathway. During TGF-β/Smad signaling activation, FUT8 increases core fucosylation of the TGF-βRI/RII complex and enhances downstream signal activity; conversely, TGF-β can induce upregulation of FUT8. The Wnt/β-catenin pathway promotes FUT8 gene transcription through activation of TCF/LEF TFs, and FUT8 in turn regulates β-catenin expression levels, although the precise mechanism remains unclear. Finally, the GPR81 complex and Cav-1 can also upregulate FUT8 by activating Wnt/β-catenin signaling. Created with BioRender.Com. EGFR, epidermal growth factor receptor; FUT8, fucosyltransferase 8; TCF/LEF, T-cell factor/lymphoid enhancer-binding factor; TF, transcription factor.

FUT8 modulates tumor biology by interacting with signaling molecules, which reciprocally regulate FUT8 expression and core fucosylation. This section details these interactions, focusing on E-cadherin, epidermal growth factor receptor (EGFR), TGF-β, and Wnt/β-catenin, summarized in Table 2 and Figure 2.

**Table 2:** The interaction between signaling molecules and FUT8

Signaling molecules	Tumor	Fut8 compare to normal	Mechinasm	Fuction	References
E-cadherin	Lung cancer	Unknown	Core fucosylation of E-cadherin modulates nuclear β-catenin accumulation and activates Src kinase	FUT8 enhances migratory ability	^[56]^
E-cadherin	BC	↑	FUT8 deficiency inhibits the FAK/integrin pathway	FUT8 deficiency suppresses adhesion, invasion, and migration	^[57]^
E-cadherin	SW480 cell line	↑	Unknown	FUT8 knockdown decreased E-cadherin expression and reduced migration	^[58]^
E-cadherin	SW620 cell line	↑	Unknown	FUT8 knockdown increased E-cadherin expression. The SW620 cell line showed no significant changes in adhesion or migration after slicing FUT8	^[58]^
EGFR/MET	Glioblastoma cells	↑	FUT8 activates both the MET/HGF and EGFR signaling axes	FUT8 promotes malignant behaviors, whereas FUT8 inhibition enhances temozolomide efficacy	^[59]^
EGFR	NSCLC	↑	FUT8 promotes core fucosylation of EGFR in CAFs	FUT8 fosters a malignant TME, exhibiting faster proliferation and increased invasiveness	^[60]^
EGFR	CRPC	↑	FUT8 shifts cell signaling from AR-dependent to EGFR-dependent pathways	FUT8 helps prevent castration-induced cell death	^[61]^
EGF	HCC	↑	FUT8 deficiency significantly reduces EGFR phosphorylation levels	FUT8 deficiency inhibits tumor cell proliferation	^[62]^
EGF	HNSCC	↑	EGF increases SEMA7A’s binding affinity for FUT8	FUT8 increases resistance to EGFR-targeted therapies and PD-L1-based immunotherapy	^[63]^
TGF-β receptor	BC	↑	FUT8 remodels the core fucosylation of the TGF-β receptor	FUT8 promotes invasiveness and metastasis of breast cancer cells	^[64]^
HGF/TGF-β1	HCC	↑	HGF or TGF-β1 treatment can increase FUT8 expression and upregulate FOLR1 core fucosylation	This mechanism enhances folate uptake capacity and promotes EMT	^[65]^
TGF-β1	HNSCC	↑	TGF-β1 induces FUT8-mediated glycosylation of SEMA7A primarily via EMT	FUT8 increases resistance to EGFR-targeted therapies and PD-L1-based immunotherapy	^[63]^
Wnt/β-catenin	CRC	↑	FUT8 knockdown increases intracellular β-catenin levels in SW480 and SW620 cells	Unknown	^[58]^
β-catenin	BC	↑	FUT8 deficiency reduces nuclear β-catenin accumulation	FUT8 deficiency suppresses adhesion, invasion, and migration	^[57]^
Wnt/β-catenin	HCC	↑	Cav-1 activates Wnt/β-catenin signaling and promotes TCF/LEF binding to the FUT8 promoter	This mechanism enhanced proliferation and invasion	^[32]^
Wnt/β-catenin	cervical cancer	↓	Lactate activates Wnt signaling and upregulates FUT8 via the lactate–GPR81 complex	FUT8 inhibits proliferation and migration.	^[37]^
Wnt/β-catenin	BC	↑	Fentanyl upregulates α1,6-fucosylation via the Wnt/β-catenin signaling pathway	FUT8 promotes stemness and epithelial–mesenchymal transition	^[66]^
Wnt/β-catenin	CRC	↑	LEF1-AS1 activates the Wnt/β-catenin/LEF1 pathway, thereby upregulating FUT8	This mechanism promotes proliferation, migration, and invasion	^[34]^
Integrin αvβ5	BC	↑	FUT8 regulates αvβ5-integrin-mediated cell adhesion	FUT8 promotes cellular invasion	^[67]^
CD147	ESCC	↑	Unknown	FUT8 mediates radioresistance and correlates with poor prognosis	^[68]^
CTR1	EOC	↑	FUT8 regulates phosphorylation of cDDP-resistance associated molecules	Core fucosylation of CTR1 improves cDDP based chemotherapy	^[69]^
EVs	Prostate cancer	↑	Unknown	Increased FUT8 expression reduces vesicle numbers, increases proteins associated with motility and metastasis, and alters glycans on select EV-derived glycoproteins	^[70]^
L1CAM	Melanoma	↑	FUT8 inhibits L1CAM cleavage	FUT8 facilitates invasion, tumor dissemination, and metastasis	^[31]^
MMPs	BC	↑	FUT8 deficiency reduces MMP-2 and MMP-9 expression	FUT8 suppresses adhesion, invasion, and migration	^[57]^
NCOA3	Pancreatic cancer	↑	Unknown	FUT8 increases mucin stability	^[71]^
sEVs	NSCLC	↑	NSCLC-derived sEVs abundantly express core-fucosylated proteins	Unknown	^[72]^
SOD3	NSCLC	↑	FUT8 maintains SOD3 secretion and enzymatic activity	FUT8 suppresses growth of NSCLC cells	^[73]^
HSP90/MUC1	HCC	↑	FUT8 promotes HSP90 core fucosylation, enhances Hsp90–MUC1 binding, and activates the JAK1/STAT3 cascade	FUT8 promotes proliferation, aggressiveness, and tumorigenesis	^[41]^
PI3K/AKT	iCCA	↑	FUT8 induces the malignant phenotypes via PI3K-AKT pathway	FUT8 promotes migration, invasion and proliferation	^[49]^
TNF/NF-κB2	OS	↓	FUT8 deficiency decreases TNFR core fucosylation and activates the NF-κB2 signaling pathway	FUT8 decreases mitochondria-dependent apoptosis	^[36]^
PSGL1	ICC	↑	The synthetic retinoid sulfarotene inhibits FUT8 activity by binding to Ser245, Asn247, and Ile242	Core fucosylation of PSGL1 is an important factor in cytoskeletal organization	^[74]^

AR, androgen receptor; CAFs, cancer-associated fibroblasts; cDDP, cellular transport of cisplatin; CTR1, copper transporter 1; EGFR, epidermal growth factor receptor; EOC, epithelial ovarian cancer; ESCC, oesophageal squamous cell carcinoma; EVs, extracellular vesicles; FUT8, fucosyltransferase 8; Hsp90, heat shock protein 90; MMPs, metalloproteinases; NSCLC, non-small cell lung cancer; PSGL1, P-selectin glycoprotein ligand 1; sEVs, small extracellular vesicles; SOD3, superoxide dismutase 3.

### E-cadherin

E-cadherin, a type I calcium-dependent adhesion protein, mediates intercellular adhesion, and its downregulation drives tumor proliferation, invasion, and loss of polarity^[75]^. Inhibition of FUT8 overactivation in esophageal cancer stem cells may impede the metastatic potential of esophageal cancer cells by downregulating E-cadherin and stabilizing E-cadherin contact inhibition^[76]^. In a human lung giant cell carcinoma cell line, FUT8-mediated core fucosylation of E-cadherin impaired calcium-dependent cell-to-cell adhesion involving E-cadherin, and computer simulation studies confirmed that this phenomenon was due to significant disruption of the three-dimensional conformation of the *N*-glycosylation chain of E-cadherin by core fucosylation^[77]^. However, another study in lung giant cell carcinoma cell lines suggested that FUT8-mediated inhibition of EMT might contribute to an alternative tumor outcome, specifically, FUT8-mediated nuclear fucosylation of E-cadherin inhibited Src kinase activation and blocked intranuclear accumulation of β-catenin, and in addition, inhibition of the Src-Akt-GSK-3β pathway occurred simultaneously, and the combined effect of the two pathways led to inhibition of EMT^[56,78]^. In BC, FUT8 deficiency suppresses the FAK/integrin signaling pathway by inhibiting core fucosylation of E-cadherin, thereby reducing the migratory capacity of BC cells^[57]^. Moreover, the tumor stage can also affect FUT8-regulated E-cadherin expression, which dynamically influences the EMT process in CRC. Compared with the SW620 cell line, FUT8 inhibition in SW480 cells reduced E-cadherin expression and promoted EMT^[58]^. In conclusion, existing studies suggest that FUT8 may influence cell–cell adhesion by altering the expression and three-dimensional conformation of E-cadherin. The impact of E-cadherin core fucosylation on downstream signaling pathways is also gradually being gradually revealed. FUT8’s effects on E-cadherin are crucial for cancer cell migration and vary according to the type and stage of the cancer.

### EGFR

The EGFR is a key receptor with tyrosine kinase activity that plays an important role in cell growth and differentiation. EGFR exerts its function primarily through ligand binding, which triggers receptor dimerization and activates a series of intracellular signaling pathways, thereby regulating gene expression, cell proliferation, and survival. In cancer, EGFR is often overexpressed or mutated, leading to abnormal activation, particularly in lung cancer, glioblastoma, and CRC^[79]^. EGFR-targeted therapies have proven to be effective in treating these tumors. Additionally, researchers have gradually discovered that the *N*-glycosylation pattern of EGFR can influence its function. Receptor tyrosine kinases (RTKs) are key signaling molecules in the EGFR signaling pathway. Kuo-Chen Wei and colleagues found that FUT8 silencing in glioblastoma reduced the phosphorylation of MET, EPHA1, and RYK. In contrast, FUT8 overexpression enhanced the phosphorylation of various RTKs including ErbB2, FGFR1, FGFR2, AXL, and MET. Moreover, FUT8 upregulation promotes core fucosylation of EGFR and induces transactivation of EGFR via the HGF/MET pathway^[59]^. In castration-resistant prostate cancer (CRPC) cells overexpression FUT8, Höti *et al* found that the protein level of EGFR was significantly elevated. EGFR overexpression drives cancer cell growth and proliferation by activating the RAS, RAF, MAPK, and JNK1 proteins, and for the first time, they revealed that the conversion of AR signaling to EGFR signaling contributes to CRPC formation^[61]^. In mice with HCC, Fut8-deficient cells showed a reduced response to EGF. This suggests a close relationship between EGFR’s spatial structure of EGFR and its core fucosylation, with FUT8 deficiency potentially impairing the formation of EGFR dimers and ligand–receptor complexes, thus affecting the transmission of multiple pro-cancer signaling pathways, including Ras/MAPKK, c-Met, and Akt/mTOR^[62]^. As an important component of the tumor microenvironment, the expression of FUT8 in cancer-associated fibroblasts (CAFs) is also related to cancer malignancy. In NSCLC, FUT8 downregulation in CAFs inhibits EGFR and its downstream phosphorylation of ERK, AKT, and JAK by reducing core fucosylation of EGFR. The impact of FUT8 downregulation on CAFs and the tumor microenvironment may be one of the reasons for inhibiting cancer cell entry into the G1/S phase, thereby suppressing proliferation^[60]^. In summary, core fucosylation is the crucial structural basis for EGFR activation. FUT8 promotes cancer cell proliferation by increasing the core fucosylation of EGFR and contributes to CAF formation in the tumor microenvironment. It may also transactivate EGFR through the HGF/MET pathway, thereby increasing cancer malignancy.

### TGF-β

TGF-β is a widely expressed cytokine that regulates a variety of physiological processes, including cell proliferation, differentiation, and migration. In cancer, TGF-β signaling plays a dual role: in the early stages, TGF-β acts as a tumor suppressor by inhibiting cell proliferation and differentiation and inducing apoptosis; in the later stages, TGF-β promotes cancer progression by driving cellular transformation, EMT, invasion, and metastasis^[80]^. After TGF-β binds to its receptor, TβRII, the intracellular kinase domain of the receptor is activated. TβRII then recruits and phosphorylates TβRI to form a heterodimeric complex. The phosphorylation of TβRI by TβRII promotes the formation of a complex between SMAD2/3 and SMAD4, which then translocates to the nucleus to regulate the transcription of target genes^[81]^. Core fucosylation is an important structural basis for the activation of the TGF-β signaling pathway. In a rat peritoneal mesothelial cell (PMCs) model transfected with Fut8 siRNA, silencing FUT8 inhibited the phosphorylation of Smad2/3, thereby suppressing the activation of the TGF-β/Smad signaling pathway and weakening the EMT of rat PMCs, without affecting the expression levels of Smad2/3 and TGF-β receptors^[82]^. *In vitro*, reducing CF modification of TGF-βR in renal tubular cells also caused a loss of TGF-β/Smad2/3 signal activation^[83]^. Studies have shown that *N*-glycosylation of TβRII in LUAD and gastric cancer cell lines interferes with the binding of TGF-β1 to TβRII, leading to resistance to TGF-β signaling^[84]^. Cheng-Fen Tu and colleagues found that during TGF-β-induced EMT in BC cells, FUT8 was upregulated and increased the core fucosylation of the TGF-βRI and TβRII complex, thereby enhancing downstream signaling activity. This mechanism promotes EMT in BC cells and increases their migration and invasion capacity^[64]^. Li Jia and colleagues found that TGF-β1 upregulated FUT8 and promoted core fucosylation of FOLR1, thereby increasing cellular folate uptake and promoting EMT progression in HCC^[65]^. Therefore, the core fucosylation structure of TGF-β receptors is crucial, and its pathway activation may serve as one of the triggers for FUT8 upregulation during EMT, with the interaction mechanism between the two warranting further investigation.

### Wnt/β-catenin

The Wnt/β-catenin signaling pathway is a conserved and crucial pathway that coordinates multiple cellular signaling cascades. It plays a key role cell proliferation, differentiation, apoptosis, migration, invasion, and tissue homeostasis^[85]^. The destruction complex (DC), composed of APC, Axin, glycogen synthase kinase 3β (GSK3β), and CK1α, regulates the Wnt/β-catenin signaling pathway. When Wnt is not bound to the FZD and LRP5/6 receptors, DC phosphorylate β-catenin, leading to its ubiquitination and subsequent degradation by the proteasome. In contrast, when the FZD receptor is activated, it recruits the phosphorylated protein Dishevelled, thereby inhibiting DC activity. This inhibition results in the accumulation of β-catenin in the cytoplasm, which is partially translocated to the nucleus^[86]^. Nuclear β-catenin activates TCF/LEF, promoting the transcription of FUT8. Existing studies indicate that in OSCC, CRC, HCC, cervical cancer, and NSCLC, FUT8 transcription is upregulated via this mechanism^[34,37,42,54,66]^. In contrast to most cancers, FUT8 negatively regulates the cervical cancer malignancy. Knockout of the Fut8 gene enhances the mTOR, cAMP, and MAPK signaling pathways and reduces the TNF signaling pathway, thus increasing cell migration while decreasing cell adhesion and apoptosis^[37]^. Qingjie Fan *et al* explored the relationship between the vaginal microenvironment and core fucosylation in cervical cancer cells. The Wnt/β-catenin signaling pathway is activated by the lactic acid produced by Lactobacillus spp. in the vaginal environment, forming a complex with Gpr81, which increases the core fucosylation levels of cervical squamous cells and inhibits the proliferation and migration of cervical cancer cells^[37]^. In a study on the effects of analgesics in BC, fentanyl was found to increase the expression of key molecules (p-GSK-3β and β-catenin) and target genes (Cyclin D1, CD44, VEGF, and c-Jun) in the Wnt/β-catenin signaling pathway. It also promotes the translocation of β-catenin from the cytoplasm to the nucleus, thereby activating the Wnt/β-catenin signaling pathway. This induces cancer stemness and EMT by upregulating FUT8 expression^[66]^. Cheng Zhang *et al* found that overexpression of Caveolin-1 increased β-catenin expression in both the cytoplasm and nucleus, thereby upregulating FUT8 through the activation of the Wnt/β-catenin signaling pathway and enhancing the proliferation and invasion of HCC cells^[32]^. FUT8 also affects activation of the Wnt/β-catenin pathway. In SW480 and SW620 CRC cell lines, silencing of FUT8 led to increased β-catenin expression^[58]^. In BC, FUT8 knockout reduces the accumulation of β-catenin in the nucleus^[57]^. In summary, cancer cells not only upregulate FUT8 expression by activating the Wnt/β-catenin pathway, but FUT8 can also inversely regulate β-catenin expression. The regulatory mechanisms may vary in different cancers; however, the specific molecular mechanisms remain to be clarified.

## FUT8 in tumor immunity and targeted therapies

FUT8 modulates the tumor immune microenvironment, influencing ADCC and immune checkpoint stability, and holds promise as a therapeutic target.

### Afucosylated IgG enhances ADCC

ADCC, alongside antibody-dependent cellular phagocytosis and complement-dependent cytotoxicity, mediates antibody-driven immune responses. IgG constitutes 75–80% of the total serum immunoglobulin, with IgG1 being the most abundant and playing a key role in ADCC, as well as being the primary therapeutic antibody. The Fc region of the antibody binds to the FcγRIIIa receptor on natural killer cells to activate ADCC^[87]^. Over 90–95% of serum IgG1 in the human body undergoes core fucosylation in the bifurcated *N*-glycans of its Fc region^[88]^. Core fucosylation of IgG significantly inhibits its function in ADCC, whereas IgG1 lacking core fucosylation can enhance ADCC activity 50–100 times^[89]^.

**Figure 3.**
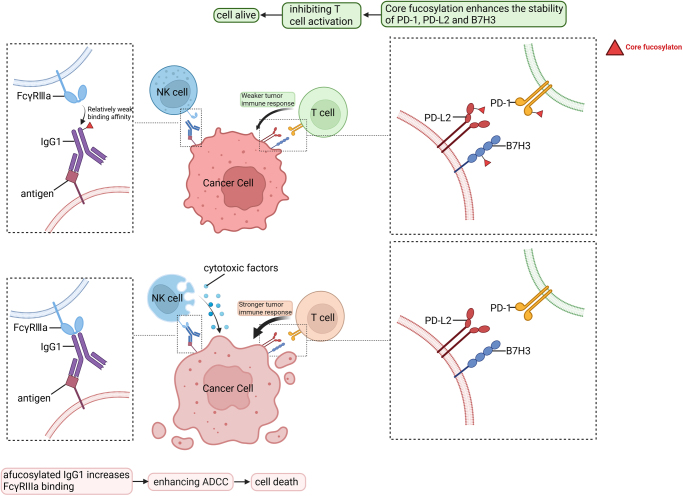
Impact of core fucosylation on the IgG1 Fc region and immune checkpoints. After IgG1 recognizes and binds to antigens on the surface of tumor cells, its Fc region interacts with the FcγRIIIa receptor on the membrane of NK cells. This interaction triggers the release of cytotoxic factors from NK cells, thereby inducing ADCC and leading to tumor cell lysis. Afucosylation of the IgG1 Fc region enhances its binding affinity to FcγRIIIa, thereby promoting tumor cell death through increased ADCC activity. PD-1, PD-L2, and B7H3 are key immune checkpoints, and current studies have shown that core fucosylation of these molecules enhances their stability, which, in turn, facilitates tumor immune evasion via immunosuppressive mechanisms. Created with BioRender.Com. ADCC, antibody-dependent cell-mediated cytotoxicity; NK, natural killer.

Compared to normally glycosylated antibodies, afucosylated anti-PD-L1 antibodies exhibit stronger FcγRIIIa-binding ability, thereby enhancing ADCC activity against PD-L1-positive cancer cells^[90]^. Therefore, in cancer therapy, removing core fucosylation from the IgG1 Fc region can enhance therapeutic efficacy, while reducing dose-related costs and side effects^[91]^(Fig. 3). The progression of CRC is associated with core fucosylation of IgG^[92,93]^. Given the significant role of core fucosylation removal in antibody therapy, inhibiting FUT8 to produce therapeutic IgG with low or no fucosylation is an important strategy. IgG1 produced by the Y2B/0 rat cell line, which naturally has low FUT8 enzyme activity, demonstrated significantly enhanced ADCC compared to antibodies produced in control Chinese hamster ovary (CHO) cells^[94]^. FUT8 knockout can also produce afucosylated antibodies, and CHO cells with knockout of both FUT8 and GMD genes can be used to produce highly efficient fully afucosylated antibodies^[95]^. Zinc finger nuclease technology and CRISPR-Cas9 gene editing have been effectively used to knock out FUT8 in CHO cell lines^[96,97]^. Simon Joubert and colleagues effectively suppressed FUT8 activity in CHO cells by co-expressing an endogenous antibody targeting FUT8, thereby reducing core fucosylation of the antibody Fc region’s *N*-glycans^[98]^. Antibodies with reduced core fucosylation were produced by targeting FUT8 mRNA with siRNA technology in CHO DG44 cells^[99]^. James M. Termini and his team used adeno-associated virus to deliver FUT8 shRNA, thereby engineering the glycosylation of antibodies to enhance ADCC activity^[100]^.

Currently, several afucosylated and low-fucosylated therapeutic antibodies have been approved for clinical use, and many others have been used in clinical trials^[101]^. Among them, some afucosylated antibodies have been approved by the Food and Drug Administration/EU^[101]^. Mogamulizumab (POTELIGEO®) was the first glycoengineering antibody to be approved. It is a humanized afucosylated monoclonal antibody that targets C–C chemokine receptor 4 (CCR4) for the treatment of relapsed or refractory CCR4-positive adult T-cell leukemia/lymphoma. The successful approval of mogamulizumab (POTELIGEO®) marks the beginning of the era of glycoengineered antibodies^[102]^. Obinutuzumab (GA101 or Gazyva®) is an anti-CD20 antibody that reduces fucosylation by co-expressing GnT-III and αManII in CHO cells, used in combination therapy for chronic lymphocytic leukemia and follicular lymphoma^[103]^. J6M0-mcMMAF (GSK2857916) is a novel humanized afucosylated antagonistic anti-BCMA antibody-drug conjugate that specifically targets and kills bone marrow cells by binding to BCMA^[104]^. JNJ-61186372 targets EGFR and c-Met and is designed to have less than 10% fucosylation. This modification enhances ADCC, thereby increasing its cytotoxicity in NSCLC cells^[105]^.

### Core fucosylation of immune checkpoints promotes immune evasion

As research has deepened, the role of core fucosylation in promoting immune evasion has become widely recognized. Immune checkpoint molecules, as important regulators of cancer immune evasion, are closely related to immune evasion mechanisms mediated by their core fucosylation (Fig. 3). On the surface of programmed cell death protein 1 (PD-1)-positive cells, N49 and N74 are the primary core fucosylation *N*-glycosylation sites. Inhibition of FUT8 can enhance T-cell activity by reducing the cell surface expression of PD-1^[106]^. In NSCLC, knocking out FUT8 increases the ubiquitination of PD-1, leading to its degradation in the proteasome, thereby enhancing the anticancer activity of cytotoxic T lymphocytes^[107]^. Chih-Wei Chu and colleagues found that in NSCLC, the concentration of fucosylated PD-1 in the blood is associated with disease staging, and the antibodies pembrolizumab and cemiplimab preferentially bind to the asparagine N58 site in core fucosylated PD-1^[108]^. PD-L2 is closely related to core fucosylation^[109]^. In head and neck squamous cell carcinoma (HNSCC), the EGFR/STAT3 signaling pathway recruits FUT8 to promote fucosylation of PD-L2. PD-L2 interacts with EGFR in an FUT8-dependent manner, and this mechanism enhances resistance to EGFR-targeted therapy by promoting abnormal regulation of the EGFR signaling pathway^[109]^. B7-H3 (CD276), an immune checkpoint molecule in the B7 superfamily, is closely associated with patient prognosis. In triple-negative breast cancer (TNBC), *N*-glycosylation of the NXT motif on B7-H3 maintains protein stability and immunosuppressive function. FUT8 catalyses the core fucosylation of B7-H3, which is crucial for its high expression. Knockout of FUT8 can reverse the immunosuppressive function mediated by glycosylated B7-H3 and enhance T-cell proliferation and activation^[110]^. In metastatic CRC,deglycosylation of B7-H3 at the N104 site induces the binding of heat shock protein family A member 8 (also known as HSC70) to its SLRLQ motif. This interaction promotes the degradation of B7-H3 via chaperone-mediated autophagy^[111]^.

In addition to PD-1, PD-L2 and B7-H3, PD-L1, B7-H4, CTLA-4, TIM-3, and LAG-3 are also important immune checkpoint molecules. *N*-Glycosylation has been found to play a significant role in these molecules, but there are no published reports on the effect of core fucosylation on their function. PD-L1 undergoes extensive *N*-glycosylation in tumor cells, and glycosylation at N192, N200, and N219 contributes to PD-L1 protein stability. Specifically, *N*-glycosylation of PD-L1 antagonizes its interaction with GSK3β, thereby inhibiting the β-TrCP-mediated, phosphorylation-dependent proteasomal degradation of PD-L1^[112]^. In LUAD, both FUT8 and PD-L1 are markedly overexpressed and significantly associated with reduced overall survival^[107]^. B7-H4 has five main *N*-glycosylation sites (N112, N140, N156, N160, and N255), that enhance the stability of the B7-H4 protein^[113]^. In CTLA-4, the number of *N*-glycans is relatively low, but they play an important role in the endocytosis of CTLA-4^[114]^. Computer molecular simulations have shown that *N*-glycosylation at N78 of T-cell immunoglobulin and mucin domain-containing 3 (TIM-3) adopts a folded conformation that, through hydrogen-bond formation and steric hindrance by the glycan, enhances the stability of small-molecule antagonist binding to TIM-3^[115]^. *N*-Glycosylation does not affect TIM-3 ligand-binding activity in CD4⁺CD25⁺ T cells; however, the interaction with Galectin-9, TIM-3’s canonical ligand, is highly dependent on TIM-3’s *N*-glycan structures, and deglycosylation abrogates this interaction^[116]^. Multiple *N*-glycosylation sites were present within the D2–D4 domains of lymphocyte activation gene-3 (LAG-3). The N184 glycan in the D2 domain makes direct contact with the R192 residue, significantly increasing the binding interface and the stability of the LAG-3 homodimer^[117,118]^. Therefore, the core fucosylation of PD-L1, B7-H4, CTLA-4, TIM-3, and LAG-3 may be closely related to their stability and immunosuppressive functions, and represents one of the forthcoming directions for research.

### Therapeutic potential of FUT8 inhibitors

Given that GDP-l-fucose is a common substrate in fucosylation reactions, analogs targeting GDP-l-fucose and its biosynthetic pathway have become the main research focus for nonselective FUT inhibitors. Examples include 2-fluoro-l-fucose (2F-Fuc), GDP-2F-Fuc, and 2-fluorofucose (SGN-2FF). SGN-2FF is an orally bioavailable small-molecule inhibitor that functions through the salvage synthesis pathway. In a 2021 first-in-human, first-in-class, phase I study of advanced solid tumors, SGN-2FF demonstrated preliminary antitumor activity, but was associated with thromboembolic events^[119]^. In addition, Pijnenborg *et al* designed a novel FUT inhibitor, fluorinated rhamnosides, which competitively inhibit GMDS and target de novo biosynthesis of GDP-fucose^[120]^. In recent years, selective FUT8 inhibitors have provided new strategies for cancer treatment and have become a key target in the development of targeted therapeutics. The team led by Wang M developed a highly selective small-molecule FUT8 inhibitor, FDW028, which specifically targets FUT8 and has demonstrated significant efficacy against mCRC both *in vitro* and *in vivo*^[111]^. Furthermore, Manabe *et al* developed FUT8-selective inhibitors using a high-throughput screening system. These inhibitors exhibit good membrane permeability and suppress EGFR phosphorylation and T-cell signaling in HepG2 cells^[121]^.

Given the critical role of core fucosylation in immune checkpoints, FUT8-selective inhibitors, a novel class of targeted therapeutic agents, show promising development potential. In a TNBC mouse model, the combination of 2F-Fuc and an anti-PD-L1 antibody enhanced therapeutic efficacy, possibly due to the degradation of fucosylated B7-H3^[110]^. Therefore, FUT8 inhibitors may exert synergistic effects with other immunotherapies. First, FUT8 inhibitors can influence multiple cancer-related molecular pathways, such as E-cadherin, EGFR, and TGF-β pathways, thereby attenuating cancer malignancy. Second, FUT8 inhibitors may reduce the expression levels of immune checkpoint molecules, including PD-1, PD-L2, and B7-H3, thereby decreasing tumor resistance to immune checkpoint inhibitors. They may also reshape the TME, enhance T-cell infiltration and function, and act synergistically with checkpoint inhibitors to promote anti-tumor immunity. However, considering the widespread distribution of FUT8 in normal human tissues, optimizing drug delivery systems to minimize off-target effects may be the key to improving the safety and efficacy of FUT8-targeted therapies.

## FUT8 as a cancer biomarker

Early cancer diagnosis and appropriate treatment plans can extend patients’ survival and improve their quality of life. Abnormal core fucosylation structures are characteristics of glycosylation changes in cancers. Detecting these abnormal core fucosylation structures or FUT8 content can help enhance cancer screening and diagnostic markers. Although FUT8 alone cannot diagnose specific cancers, combining core fucosylation structures with other biomarkers increases the specificity and sensitivity of cancer diagnosis and provides valuable clues for distinguishing between different cancer stages and prognoses. Alpha-fetoprotein (AFP) was first reported in 1956 and has been widely used in clinical practice as the most classic biomarker for diagnosing HCC^[106]^. Research has shown that AFP-L3 increases the specificity and sensitivity of AFP in diagnosing HCC. Abnormal elevation of AFP-L3 in HCC patients’ serum is closely related to the loss of function of the carrier protein vesicular integral membrane protein 36^[122]^. Yutaka Okagawa and colleagues found that the elevated AFP-L3 in HCC is due to the activation of the FUT8 gene by wild-type p53, which prevents the potential drug resistance caused by low p53 expression, thus allowing targeted drug treatment for AFP-L3-producing HCC^[43]^. This study indirectly reflects gene expression and drug resistance through biomarkers, expanding the application of AFP-L3 from diagnosis to treatment. In prostate cancer, the core fucosylation pattern of Prostate-Specific Antigen (PSA) helps identify high-grade prostate cancer and shows a good ability to differentiate it when combined with sialylated PSA^[123]^. Haptoglobin (Hp), which is mainly secreted by the liver, has abundant *N*-glycosylation sites, making it a potential glyco-biomarker for various cancers. Abnormal elevation of fucosylated Hp in the serum of patients with cancer was first discovered in 1987^[124]^. Recent studies have shown its diagnostic and prognostic value in cancers such as prostate cancer, advanced renal cell carcinoma, cholangiocarcinoma and HCC^[125-129]^.

With the continuous advancement of sequencing and analytical technologies, bioinformatics is playing an increasingly important role in cancer diagnosis and treatment. The application of techniques such as single-cell sequencing and enrichment analysis can reveal the role of FUT8 in the tumor microenvironment, thereby improving the prediction of patient prognosis. High-throughput transcriptomic sequencing analysis confirmed that FUT8 can serve as a key biomarker for determining prognosis. Tapak *et al* used a deep learning-based model for oral cancer prognosis, in which FUT8 mRNA expression is an important prognostic markers^[130]^. Another study revealed that FUT8 is significantly upregulated in advanced oral cancer compared with the early stages of the disease^[131]^. GEO data also confirmed that FUT8 expression is closely related to the progression of LUAD and invasiveness of colon cancer cells, and its expression can serve as an independent prognostic factor^[132,133]^. Moreover, bioinformatics can be used to predict FUT8-related pathways and their interactions with other cells. Xin Zhu and colleagues confirmed that in ccRCC patients, FUT8 expression was higher in endothelial and fibroblast cells, and patients with higher expression of core fucosylation-related genes had lower immune cell activity and poor overall survival^[30]^. Fengzhou Li and colleagues confirmed that overexpression of FUT8 in CAFs promoted the proliferation and invasion of NSCLC, and enrichment analysis revealed that EGFR downstream signaling molecules are closely related to FUT8 expression^[60]^. Xuyao Xu and colleagues constructed an ovarian cancer (OC) prognosis gene model using glycosyltransferases, confirming that FUT8 is associated with good prognosis and speculating that FUT8-mediated core fucosylation may limit the sialylation of cancer^[134]^. In summary, bioinformatic technologies are gradually unveiling the position and role of FUT8 in cancer, providing directions for further experimental verification.

In addition, given that FUT8 expression is significantly increased in most cancers, integrating pan-cancer core fucosylation secretion characteristics through omics analysis and identification of circulating biomarkers is expected to become a key strategy for early cancer diagnosis, treatment, and prognosis prediction. Recent studies have shown that core fucosylation is a major feature of the therapy-induced secretome, which is related to resistance and recurrence mechanisms^[135]^. Although predictions of FUT8 and its mediated core fucosylation-related pathways through modern high-throughput sequencing and single-cell technologies are relatively few, they also exhibit enormous predictive potential.

## Challenges and future directions

In HCC and CRC, increased expression of FUT8 is associated with aggressiveness of malignancy. However, p53, a prominent tumor suppressor gene, positively regulates the transcription of FUT8 mRNA. Noda and colleagues showed that FUT8 protein expression was significantly associated with better disease-free survival only in p53-negative tumors, suggesting that FUT8 expression may have a functional impact on tumors depending on p53 status^[44]^. In the future, a more comprehensive exploration of the relationship between FUT8 and p53 and its effect on cancer malignancy could become an important research direction.

FUT8, a fucosyltransferase, is encoded by the FUT8 gene. however, it is not the only product. cFUT8 and FUT8-AS1 arise from the reverse splicing of pre-mRNA and reverse transcription of FUT8, respectively. Although the existing research is relatively limited, these studies suggest that both positively regulate the transcription and translation of FUT8. Among them, cFUT8, by binding to miRNA, not only affects FUT8 mRNA but also influences the expression of other RNAs within tumor cells. In HCC, m6A-modified cFUT8 is transferred to the cytoplasm via YTHDC1 and, through competitive binding with miR-552-3p, upregulates CHMP4B mRNA, ultimately accelerating the malignant behavior of HCC^[136]^. cFUT8 is significantly upregulated in NSCLC and HCC and can serve as a potential diagnostic circRNA in CRC patients^[137-139]^. In contrast to its pro-cancer role in the above cancers, cFUT8 directly binds to miR-570-3p to inhibit of bladder cancer metastasis^[140]^.

Research on the molecular mechanism of FUT8-AS1 remains sparse, but bioinformatics studies indicate its association with glioblastoma, OC, and pediatric acute lymphoblastic leukemia^[141-143]^. Notably, in melanoma, FUT8 was upregulated, while FUT8-AS1 was downregulated, indicating significant differences in the transcriptional initiation mechanisms of FUT8 protein and FUT8-AS1, despite both being products of the FUT8 gene^[31,55]^.

Existing studies indicate that, in most cancers, the degree of malignancy is positively correlated with FUT8 expression; exceptions are observed in gastric cancer, OS, and cervical cancer. Unfortunately, current molecular biology research cannot fully explain the basis of this dual role. However, the divergent malignant behaviors of FUT8 across tumor types result from the interplay of multiple factors. These may be attributed to four aspects: first, differences in signaling-pathway dependency. First, differences in signaling pathway dependency play a crucial role. Fan Qingjie’s group demonstrated via transcriptomic analysis that FUT8 knockout in cervical cancer enhances mTOR, cAMP, and MAPK signaling while reducing TNF signaling, thereby promoting proliferation and migration and inhibiting apoptosis^[37]^. In OS, FUT8 mediates core fucosylation-dependent suppression of the TNF/NF-κB2 pathway; thus, FUT8 downregulation releases NF-κB2 and drives tumor progression^[36]^. In NSCLC, BC, and colon cancer, conformational alterations of E-cadherin facilitate EMT^[41,57,58]^. In contrast, HCC appears to be more reliant on pro-proliferative signals, such as STAT3 and EGFR: FUT8 upregulation strengthens the Hsp90/MUC1/STAT3 cascade and activates downstream EGFR pathways^[41,62]^. Thus, when a tumor depends predominantly on NF-κB survival signaling, FUT8 exerts a tumor-suppressive effect; if it relies on STAT3/E-cadherin/EGFR-mediated pathways, FUT8 may promote tumor growth. Second, variations in primary FUT8 targets among tissues. TNF receptors are likely the key substrates in OS and cervical cancer cells^[36,37]^. Colon cancer revealed GMDS mutation which impairs fucosylation that leads to immune evasion^[144]^. Such target heterogeneity implies that alterations in FUT8 affect distinct signaling networks across cancer types, resulting in divergent roles in tumor progression. Third, differences in tumor microenvironment and cytokine regulation affecting FUT8 substrates. For example, lactic acid produced by vaginal lactobacilli in the cervical cancer microenvironment upregulates FUT8 via GPR81 signaling^[37]^. Within CAFs, FUT8 modulates EGFR signaling to drive NSCLC progression^[60]^. Moreover, cytokines – such as IL-6, TNF-α, TGF-β, and EGF – in the extracellular milieu directly influence downstream JAK/STAT3, NF-κB, and Akt/mTOR activation, thereby shaping cancer cell behavior. These contextual factors cause identical FUT8 levels to exert different effects on distinct microenvironments. Fourth, there were differences in the regulatory control of FUT8 expression. FUT8 expression is governed by various TFs and ncRNAs across different tumor types. Studies have shown that TGIF2, p-STAT3, TCF/LEF, and p53 can initiate FUT8 transcription, and ncRNA-mediated regulation of FUT8 has been identified in multiple cancers. Overall, FUT8 expression is closely linked to tumor malignancy, yet the precise reasons for its divergent effects across cancer types remain unclear. Future multi-omics integrative analyses, enabled by high-throughput technologies and bioinformatics, will be essential to elucidate the regulatory networks governing FUT8 and clarify its mechanistic roles in cancer.

Currently, we face several unresolved questions and research directions. At the molecular-mechanistic level: the influence of p53 status on FUT8 transcription remains unclear; the mechanisms by which FUT8 regulates the β-catenin pathway require further elucidation; the effects of core fucosylation on the stability and expression levels of certain immune checkpoints need to be defined; and the underlying basis for FUT8’s dual roles in cancer warrants deeper investigation. At the clinical-application level: expansion of afucosylated antibody types and optimization of their manufacturing processes; combined use of FUT8 inhibitors with targeted agents and targeted-drug delivery systems, and their performance in preclinical and clinical studies; and the application of core fucosylation as a biomarker or within predictive models – all represent critical links between FUT8 research and clinical translation. Nonetheless, this review has several limitations. Glycosylation is an important and complex post-translational modification. While this work provides a detailed account of FUT8 and its mediated core fucosylation in cancer, it does not clarify the relationship between FUT8 and other glycosyltransferases. Moreover, FUT8 is also closely implicated in other diseases, such as chronic obstructive pulmonary disease, liver fibrogenesis, atherosclerosis, and Alzheimer’s disease. Investigating non-neoplastic diseases in concert may facilitate a deeper understanding of FUT8’s mechanisms of action.

## Conclusion

FUT8 regulates cancer biology through core fucosylation, modulating signaling pathways, immune responses, and tumor behavior. Its interactions with E-cadherin, EGFR, TGF-β, and Wnt/β-catenin drive malignancy, while its role in ADCC and immune checkpoint stability highlights therapeutic potential. FUT8 inhibitors and afucosylated antibodies offer promising strategies, and its biomarker potential could transform diagnostics. Despite challenges, such as tissue-specific duality and delivery optimization, FUT8 holds immense promise for advancing cancer treatment and prognosis.

## Data Availability

Not applicable.
